# AID–RNA polymerase II transcription-dependent deamination of IgV DNA

**DOI:** 10.1093/nar/gkz821

**Published:** 2019-09-30

**Authors:** Phuong Pham, Sohail Malik, Chiho Mak, Peter C Calabrese, Robert G Roeder, Myron F Goodman

**Affiliations:** 1 Department of Biological Sciences, University of Southern California, Los Angeles, CA 90089, USA; 2 Laboratory of Biochemistry and Molecular Biology, The Rockefeller University, New York, NY 10065, USA; 3 Department of Chemistry, University of Southern California, Los Angeles, CA 90089, USA

## Abstract

Activation-induced deoxycytidine deaminase (AID) initiates somatic hypermutation (SHM) in immunoglobulin variable (IgV) genes to produce high-affinity antibodies. SHM requires IgV transcription by RNA polymerase II (Pol II). A eukaryotic transcription system including AID has not been reported previously. Here, we reconstitute AID-catalyzed deamination during Pol II transcription elongation in conjunction with DSIF transcription factor. C→T mutations occur at similar frequencies on non-transcribed strand (NTS) and transcribed strand (TS) DNA. In contrast, bacteriophage T7 Pol generates NTS mutations predominantly. AID-Pol II mutations are strongly favored in WRC and WGCW overlapping hot motifs (W = A or T, R = A or G) on both DNA strands. Single mutations occur on 70% of transcribed DNA clones. Mutations are correlated over a 15 nt distance in multiply mutated clones, suggesting that deaminations are catalyzed processively within a stalled or backtracked transcription bubble. Site-by-site comparisons for biochemical and human memory B-cell mutational spectra in an IGHV3-23*01 target show strongly favored deaminations occurring in the antigen-binding complementarity determining regions (CDR) compared to the framework regions (FW). By exhibiting consistency with B-cell SHM, our *in vitro* data suggest that biochemically defined reconstituted Pol II transcription systems can be used to investigate how, when and where AID is targeted.

## INTRODUCTION

Activation-induced deoxycytidine deaminase (AID) plays a central role in the generation of antibody (Ab) molecules that bind with high-affinity to invading antigen (Ag) molecules, a vital step in defending against infection in eukaryotes (1). AID is expressed selectively in immune B-cells (2), and initiates somatic hypermutation (SHM) and class-switch recombination (CSR) (3–5) by deaminating C→U during transcription of Ig-variable (V) and Ig-switch (S) region DNA (6–8). Subsequent replication of U•G mispairs causes mutations at AID-deaminated G:C sites. Repair of U•G with either base-excision repair (BER) or post-replication mismatch repair (MMR) pathways, involving error-prone DNA polymerase η (pol η), results in mutations at A:T sites within IgV repair tracts, or causes dsDNA breaks which serve as foci for initiating CSR (3–5,9).

Our paper is focused on the biochemical mechanisms of transcription-dependent dC deamination by AID acting in conjunction with human RNA polymerase II (Pol II). In the absence of other factors, AID has been shown to deaminate dC solely on single-stranded DNA (ssDNA) (10,11). Previous biochemical experiments using T7 RNA polymerase (T7 Pol) have shown that AID acts preferentially on the NTS during transcription (11–13), in accord with the stringent substrate specificity of AID. Similarly, AID-catalyzed deamination during transcription in *Escherichia coli* occurs primarily on the NTS (13,14). Presumably, the NTS within a moving or stalled transcription bubble is fully accessible to AID, while the hybrid RNA–DNA TS is largely inaccessible.

However, SHM *in vivo* shows no strand bias. There are roughly equal numbers of mutations on NTS and TS DNA of IgV in normal mice (15) and in uracil DNA glycosylase (*ung*^−/−^) deficient mice (16,17). Strand bias was not observed for an actively transcribed hypermutating GFP gene in fibroblasts when AID is overexpressed (18). Consistent with these observations, a study of DNA structure at IgV_H_ regions in human B cell lines undergoing SHM showed multiple ssDNA patches, ∼11 nt long, on both NTS and TS (19). These ssDNA patches were suggested to represent ssDNA exposed in transcription bubbles, because they were observed only in IgV DNA that had not undergone deproteinization (19).

Based on subsequent studies with T7 Pol, several mechanisms were proposed for TS dC deamination: (i) bi-directional transcription at IgV and IgS regions (20); (ii) recruitment of an exosome complex to target AID to the TS strand in the transcription bubbles (21); (iii) the involvement of supercoiled DNA or perhaps other chromatin structural elements during transcription of IgV and IgS (22). Currently, there are no biochemical data showing how AID catalyzes deamination in a Pol II transcription model system.

In contrast to T7 Pol, transcription elongation of eukaryotic Pol II is regulated by promoter-proximal pausing and subsequent release of paused Pol II into an elongation phase (23). At least two transcription factors, a two subunit DSIF (Spt4/Spt5) and a four subunit negative elongation factor (NELF) have been shown to be involved in Pol II pausing (24,25). The conversion of paused Pol II into a transcription elongation mode requires phosphorylation of DSIF, NELF and the C-terminal domain (CTD) of the large Pol II subunit by P-TEFb kinase-transcription factor (26). During the elongation phase, DSIF remains bound to Pol II as it moves, and is likely to serve as a key player in supporting the function of other transcription elongation and pausing factors such as Pol II-associated factor 1 complex (PAF1C) (27–29). It is therefore timely to begin investigating AID function in a ‘minimal’ transcription elongation system comprised of human Pol II and DSIF during transcription of an IgV target gene.

AID interacts directly with Pol II (30), and AID genome-wide occupancy appears strongly correlated with Pol II distribution (31). At least two transcription elongation factors, the Spt5 component of DSIF (32) and PAF1C (33), have been shown to interact directly with AID during CSR. Accumulation of Spt5, along with AID and Pol II was observed in IgV and IgS regions of germinal B-cells (34), indicating its involvement in SHM and CSR. It has been suggested that AID-Spt5 interactions could be responsible for targeting AID to stalled transcription bubbles at IgV and IgS regions (32,34,35). Currently, there are no biochemical data showing how AID catalyzes deamination in a *bona fide* mammalian Pol II transcription model system or how Spt5 modulates AID recruitment or influences deamination during IgV or IgS transcription.

Here, we report the biochemical reconstitution of a transcriptional elongation system using purified human proteins, AID, Pol II and DSIF, to investigate mechanisms of AID scanning and catalysis during transcription of IGHV3-23*01, which is the most commonly used variable region during normal immune responses (36,37) and in chronic lymphocytic leukemia (38). A maximum depth sequencing analysis (39) is used to investigate AID NTS and TS deamination frequencies, and to compare biochemical and B-cell spatial deamination spectra.

## MATERIALS AND METHODS

### DNA substrates

DNA and RNA oligonucleotides were purchased from Integrated DNA Technologies (IDT) and purified by 12% denaturing polyacrylamide electrophoresis.

### Preparation of cloned transcribed strand (TS) and non-transcribed strand (NTS) ssDNA

To minimize mutation background associated with chemical DNA synthesis, TS and NTS ssDNA sequences of IGHV3-23*01 (Supplemental Figure S1) were amplified by PCR and cloned into Pvu II sites of M13mp3 phage vector and verified by Sanger DNA sequencing. Circular ssDNA M13mp2 (100 nM) containing the TS or NTS sequence was prepared from purified M13 phage and annealed with two 20 nt primers (400 nM each, 5′CGGTTCACAGCTGATTGCCC3′ and 5′ATTACGCCAGCTGGCAGTAC3′ for the TS strand; 5′GTACTGCCAGCTGATTGCCC3′ and 5′ATTACGCCAGCTGTGAACCG3′ for the NTS) in 1 ml of Pvu II restriction buffer. Following Pvu II restriction digestion at 37°C for 2 h, digested DNAs were separated by preparative 10% denaturing polyacrylamide gel electrophoresis. ssDNA bands corresponding to 203 nt TS and NTS strands were cut out and DNAs were extracted, concentrated to 100 nM in 10 mM Tris (pH 8.5) and stored at −20°C.

### Human AID preparation

A carboxy (C)-terminal hexa-His-tagged GST-AID variant (40) was expressed in Sf9-infected insect cells and purified and activated as follows. Infected Sf9 cells were suspended in lysis buffer containing 20 mM HEPES pH 8.0, 1 M NaCl, 1 mM DTT, 10 mM NaF, 10 mM NaHPO4, 10 mM sodium pyrophosphate, 5 mM imidazole, 0.2% Triton X-100, 10% glycerol and EDTA-free protease inhibitor cocktail (Roche). The cells were lysed by sonication, and the crude lysate was cleared by centrifugation at 15 000 g for 30 min. The supernatant containing GST-AID was incubated with Ni-NTA resin (Qiagen). After extensive washing, GST-AID was eluted using the lysis buffer supplemented with 250 mM imidazole. GST-AID was activated by addition of RNase A (Qiagen) (8 μg/ml) and incubated at 30°C for 5 min to digest inhibitor RNA (10). RNase A was washed away by binding GST-AID to Glutathione Sepharose resin (GE Healthcare) for 1 h followed by extensive washing with the lysis buffer. GST-AID was eluted from the resin using an elution buffer of 10 mM Tris (pH 9.8), 500 mM NaCl, 1 mM EDTA, 1 mM DTT and 10 mM reduced glutathione. Fractions containing GST-AID were dialyzed against 20 mM Tris, pH 7.5, 50 mM NaCl, 1 mM EDTA, 1 mM DTT and 10% glycerol overnight and stored at −70°C.

### Human Pol II and DSIF preparation

Human Pol II was purified from nuclear extract derived from a HeLa cell line that stably expressed the RPB9 subunit, essentially as described (41). To generate recombinant human DSIF, the Spt4 and Spt5 subunits were co-expressed in Sf9 cells. The Spt4 cDNA was subcloned into pFastBac; Spt5 cDNA was subcloned into a pFastBac derivative that allowed the corresponding protein to be expressed as an N-terminal FLAG-tagged protein (f-Spt5). Corresponding bacmids, which were obtained through an intermediate transformation into DH10Bac, were separately transfected into Sf9 cells. The resulting baculoviruses were amplified by serial passaging. For large-scale protein production, the two amplified viruses were co-infected into Sf9 cells growing in suspension culture at relative multiplicities of infection (MOI) that favored over-expression of untagged Spt4 over f-Spt5. Infected cell nuclear extract (42) was used to affinity purify DSIF using standard methods (41). Briefly, the extract was brought to 20 mM Tris–HCl (pH 7.9 at 4°C), 20% glycerol, 0.1 mM EDTA, 300 mM KCl, 0.1% NP40, 0.5 mM PMSF and 5 mM 2-mercaptoethanol, and was supplemented further with 30 μg/ml each of pepstain A, leupeptin, and benzamidine. After incubation of the extract with M2-agarose (Sigma) for 6 h, the beads were washed first with above buffer containing 300 mM KCl and then with the same buffer containing 100 mM KCl. Recombinant DSIF was eluted from the resin by three sequential incubations with buffer containing 100 mM KCl and 0.5 mg/ml FLAG peptide. The eluates were pooled and snap-frozen in liquid nitrogen. SDS-PAGE analyses of purified Pol II and DSIF are shown in Supplemental Figure S2.

### Pol II transcription on scaffolded bubble substrate in the absence or the presence of DSIF and AID

Human Pol II elongation complex (EC) was reconstituted on the scaffolded RNA:TS:NTS bubble substrate similar to what has been described for bovine Pol II (43,44). For each reaction, Pol II elongation complex was reconstituted at 30°C in 25 μl reaction buffer (10 mM HEPES–KOH, pH 8.2, 130 mM KCl, 5 mM DTT, 8% glycerol and 20 μg of BSA/ml) by incubating 125–250 ng (250–500 fmol) of purified Pol II with the pre-annealed RNA primer and the TS strand (50–100 fmol) for 10 min. When indicated, RNA primer labeled with ^32^P using USB Optikinase (Affymatrix) was used to monitor RNA transcript synthesis. Complete formation of transcription EC was accomplished with addition of the non-templated NTS strand (100–200 fmol) and incubated for another 10 min at 30°C. When present, 50 ng DSIF (284 fmol) was added to EC and incubated for 3 min followed by the addition of 20 ng AID (425 fmol). Transcription was initiated by addition of all four rNTP (500 μM) and incubated for 1 h at 30°C. For AID deamination in the absence of Pol II, scaffold RNA:TS:NTS was assembled in the same manner as described above, without Pol II prior to addition of AID. Reactions were terminated by twice extraction with phenol:chloroform:isoamyl alcohol (25:24:1). The extracted DNA was desalted using Biospin column P6 (Biorad). The excess of unannealed ssDNA (TS and NTS) was eliminated by treatment with 10 units of exonuclease I (NEB) at 37°C for 30 min. Exo I was removed using Monarch PCR and Reaction cleanup kit (NEB) and DNA was eluted in 20 μl of 10 mM Tris (pH 8.5).

### Library construction for Illumina sequencing

Following Exo I treatment to remove ssDNA, NTS and TS strands in Pol II transcription reactions were subjected to next-generation sequencing analysis using Maximum Depth Sequencing (MDS) (39). MDS sequencing allows elimination of PCR and sequencing errors by grouping and analyzing sequence-read families with the same unique barcode identifiers (UIDs). In the first step, TS and NTS strands were barcoded with 24 random nt UIDs at the 3′ end, using 1 PCR cycle (94°C 1 min, 45°C 1 s, 72°C 2 min) and Taq DNA polymerase. After the barcode removal and PCR reaction clean-up, a linear amplification step (10 PCR cycles: 94°C 30 s, 45°C 30 s, 72°C 1 min) by Taq DNA polymerase was carried out to allow copying of deaminated dU bases. DNA from the linear amplification was further amplified by 25–30 cycles of exponential PCR amplification (98°C 10 s, 50°C 10 s, 72°C 30 s) using high-fidelity Q5 DNA polymerase (New England Biolabs). DNA primers and adaptors for TS and NTS Illumina library constructions are listed in Supplemental Table S2. NTS and TS libraries were sequenced on an Illumina MiniSeq system using 150 bp paired-end High Output sequencing reagent kit.

### RNA-seq Illumina library preparation

The Pol II transcription reaction mix including AID (see above) was extracted twice with phenol:chloroform:isoamyl alcohol (25:24:1) to remove Pol II and AID followed by incubation with 2 units of DNAse I (New England Biolabs) for 15 min at 37°C. DNAse I was removed by extracting twice with phenol:chloroform:isoamyl alcohol (25:24:1), and RNA transcripts were desalted using a Bio-Rad Biospin column P6. RNA transcripts were ligated to a 5′-adenylated, 3′-blocked ssDNA adaptor (5′-rAppGAT CGG AAG AGC ACA CGT CTG AAC TCC AG-NH_2_-3′), using T4 RNA Ligase 2-truncated KQ (New England Biolabs) at 16°C for 16 h. Ligated RNA transcripts were annealed to a NG-8 primer (5′-CAA GCA GAA GAC GGC ATA CGA GAT TCA AGT GTG ACT GGA GTT CAG ACG T-3′) and reverse transcribed using NEBNext First Strand Synthesis Enzyme mix (New England Biolabs) in a thermocycler (25°C 10 min, 42°C 15 min, 70°C 15 min). The product cDNAs were amplified 30 cycles (94°C 30 s, 45°C 15 s, 72°C 30 s) by Taq DNA polymerase (Promega) using NG-8 and NG-RNA-F (5′-AAT GAT ACG GCG ACC ACC GAG ATC TAC ACT CTT TCC CTA CAC GAC GCT CTT CCG ATC T TAT ATG CAT AAA GAC CAG-3′) as PCR primers. The RNA-seq library was purified using AMPure XP beads (Beckman Coulter) and sequenced on a MiniSeq system.

### Sequence analysis

We used the Safe-Sequencing System (45) with UIDs of length 24. We only considered those reads with fewer than 20 bases different from the reference sequence, and quality scores of at least 30 at all sites in the UID. We clustered reads with the same UID into families. We only considered those families with at least three paired reads with the same UID. At each site, the mutation frequency is calculated by dividing a numerator by a denominator. The denominator is the number of UID families that at this particular site have at least three reads with quality scores of at least 30. The numerator is the number of UID families that at this particular site have at least three reads with quality scores of at least 30 and that 95% or more of these reads have the same base that is different than the reference (so-called ‘supermutants’). The total numbers of sequenced dC template bases and scored C to T mutations for each experiment using this analysis are listed in Table 1.

**Table 1.** Pol II-dependent AID-catalyzed deamination on IGHV3-23*01 substrate

	Experiment	No. of sequenced C^a^	No. of C to T mutations	Mutation rate (× 10^−5^)^b^	Average ± SD	*t*-test^c^
**Non-transcribed strand**						
NTS-control	1	3469440	110	3.2	-	
NTS +Pol II	1	541824	15	2.8	-	
NTS + AID	1	1926144	76	3.9		
	2	5186928	191	3.7		
	3	6159120	227	3.7	3.8 ± 0.1	
NTS+ Pol II + AID	1	648576	132	20.4		
	2	4347888	1405	32.3		
	3	4694496	1787	38.1	30.2 ± 7.4	
						*P* = *0.046*
NTS + Pol II + AID + DSIF	1	4221600	2400	56.9		
	2	4561776	2310	50.6	53.7 ± 3.1	
**Transcribed strand**						
TS-control	1	5574690	180	3.2	-	
TS + Pol II	1	1922052	49	2.5	-	
TS + AID	1	3291222	192	5.8		
	2	9993060	593	5.9		
	3	5402298	228	4.2	5.3 ± 0.8	
TS + Pol II + AID	1	2713590	1573	58.0		
	2	6243336	2632	42.2		
	3	4165854	1672	40.1	46.8 ± 8.0	
						*P* = *0.208*
TS + Pol II + AID + DSIF	1	5415564	1719	31.7		
	2	4594854	1721	37.5	34.6 ± 2.9	

^a^Total number of all sequenced dC bases (48 sites on NTS strand and 66 sites on TS strand) from qualified UID family-reads (see Materials and Methods for details).

^b^Mutation rates for each experiment were calculated as the total number of C to T mutations on the NTS or TS divided by the total numbers of all sequenced C templates on the NTS or TS, respectively. When shown, average mutation rates represent average ± standard deviation.

^c^ *P* values from two-tailed Student's *t*-test are shown for comparison of NTS mutation rates in the absence and the presence of DSIF, and TS mutation rates in the absence and the presence of DSIF.

### Analysis of mutation correlation

We analyzed the spatial relationships between mutated C on the NTS and TS by computing the covariance of mutations deposited on each clone, $c\ ( {i,j} ) = {\langle{\rm{\delta }}{h_i}{\rm{\delta }}{h_j}\rangle}/{\sigma _i}{\sigma _j}$, where the counter ${h_i} \equiv 1$ if a mutation is found at site $i$, and 0 otherwise, and ${\rm{\delta }}{h_i} \equiv {h_i} - {\langle{h_i}\rangle}$. The brackets indicate an average over the entire clone library. To account for the inhomogeneity of the mutation frequencies on the sequence, the covariances were normalized by the standard deviations ${{\rm{\sigma }}_i} \equiv {\langle {{{( {{\rm{\delta }}{h_i}} )}^2}} \rangle ^{1/2}}$ and ${{\rm{\sigma }}_j} \equiv {\langle {{{({\rm{\delta }}{h_j})}^2}} \rangle ^{1/2}}$. By definition, any covariance involving a non-C site is zero. The diagonal elements $c( {i,i} )$ have no meaning since they do not report on the correlation between two deamination events.

To measure the correlation between mutations as a function of distance between them, we then computed the distance-dependent correlation function $C( {| {i - j} |} )$ from the covariances by summing $c( {i,j} )$ over all pairs of sites $( {i,j} )$ with the same distance between them and dividing by the number of non-zero pairs in the library. This correlation function is plotted in Figure 4 for both the NTS and the TS. A previous mathematical analysis (46) of the correlation function suggests that it should be an exponential function of distance, ${e^{ - | {i - j} |/L}}$, where the characteristic length $L$ is a measure of how mutations on the sequence are correlated as a function distance between them. Since the different C sites on the sequence have heterogeneous mutation rates, this correlation function is intrinsically noisy, but the exponential fit represents the data well.

### Data and computer codes availability

All raw experimental data, including next-generation sequencing data are available. Sequence analysis was carried using standard numerical procedures. These programs are available upon request.

## RESULTS

### Human transcriptional elongation system containing AID, Pol II and DSIF

**Figure 1.**
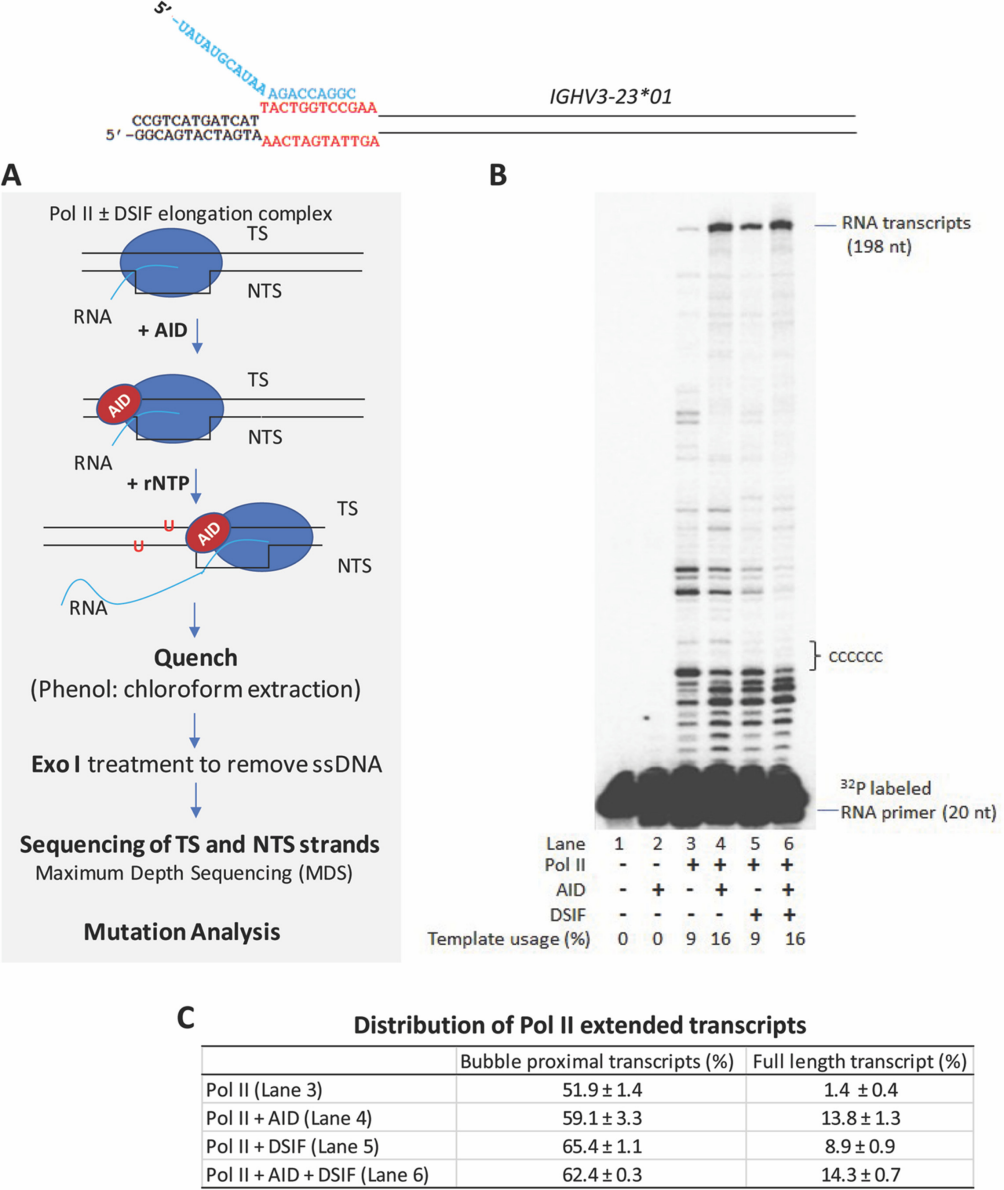
Experimental protocol used to analyze AID-catalyzed dC deamination on IGHV3-23*01 during transcription by human Pol II. (**A**) Pol II ± DSIF elongation complexes were assembled on a DNA–RNA ‘scaffolded bubble’ substrate and preincubated with AID. Transcription was initiated by the addition rNTP substrates, and the elongation reaction was performed at 30°C (Methods). Following transcription, Exonuclease I (Exo I) was added to digest ssDNA. TS and NTS DNAs were separately barcoded and subjected next-generation sequencing analysis using Maximum Depth Sequencing (MDS) (39) to assess AID-mediated dC deamination. (**B**) Transcription in the presence of AID and DSIF was visualized as ^32^P-labeled RNA primer elongation bands that extend for the full length of the IgV DNA (198 nt). A strong transcription pause region is located ∼11 nt downstream of the scaffold bubble, and is followed by six C residues on the TS, in which deaminations are observed to occur at as many as three contiguous C sites – see also Supplemental Figure S9. (**C**) Distribution of Pol II extended transcripts. Percentage (mean ± standard deviation) of scaffold bubble proximal transcripts (1–12 nt from the end of the scaffold bubble to a run of six consecutive Cs) and full-length transcript (198 nt) were quantified by GE Healthcare ImageQuant software. A sketch depicting the transcribed IgV substrate and the scaffold bubble containing a 20 nt RNA primer strand is shown at the top.

Our primary experimental system consists of purified human Pol II, AID and DSIF proteins and a DNA template that contains antigen-binding complementarity determining regions CDR1 and CDR2 of IGHV3-23*01 (Figure 1 and Supplemental Figure S1). To facilitate the transcription analysis, we have used a preformed Pol II elongation complex that allows investigation of eukaryotic transcription structures and elongation mechanisms that are independent of a promoter or proteins involved in transcription initiation (25,29,43,44,47,48). Transcription is initiated at the 3′OH end of a synthetic RNA primer annealed to the TS strand within a 12 base pair (bp) ‘scaffold bubble’ (Figure 1A). Since DSIF (Spt4/Spt5) has an established role as an elongation factor (24) and in view of a previous report of a role for Spt5 in AID-Pol II interactions (32), we first measured AID-catalyzed dC deamination with Pol II and then evaluated how DSIF might impact AID activity.

**Figure 2.**
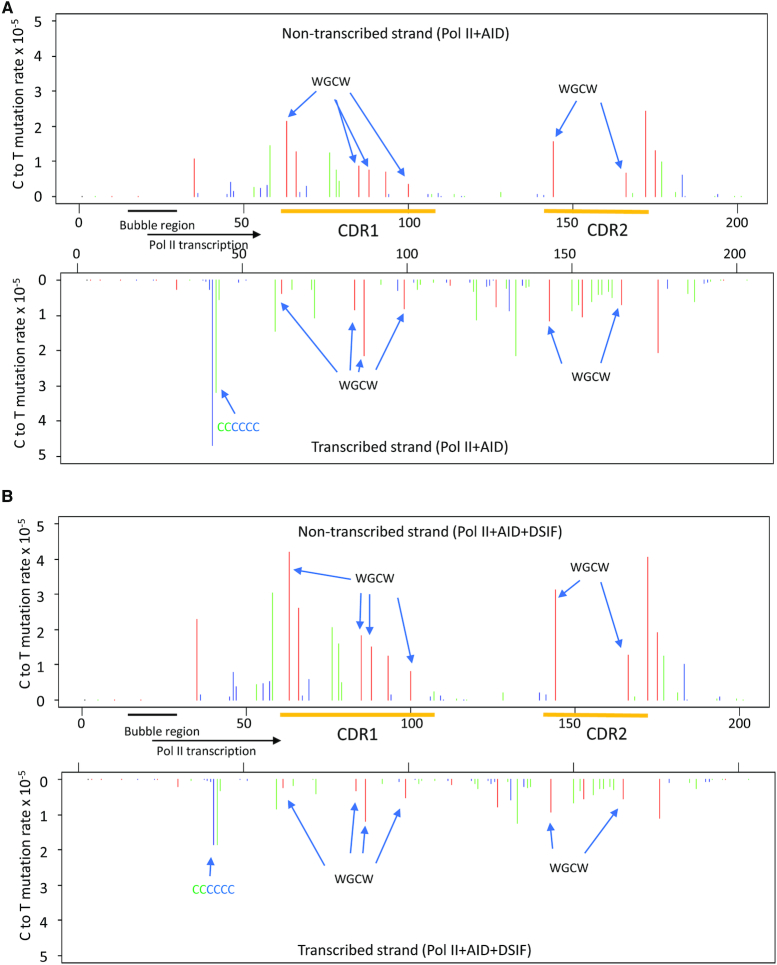
AID-catalyzed dC NTS and TS deamination spectra on IGHV3-23*01. (**A**) AID deamination spectra for the NTS (top) and TS (bottom) during transcription of IgV DNA by Pol II. Deaminations are detected as C → T mutations at C template sites determined by MDS sequencing. The C → T mutation rate is shown at each dC site in the IgV target sequence (5′WRC hot-motifs, *red* bars, 5′SYC cold-motifs, *blue* bars; all other motifs containing a C site, *green* bars). The mutation rate at each site on NTS or TS was calculated as numbers of scored C to T mutations divided by total numbers of all sequenced Cs at a given position. (**B**) AID deamination spectra for the NTS (top) and TS (bottom) strands during transcription of IgV DNA by Pol II + DSIF. In A and B, preferred overlapping hot motifs (WGCW) in IGHV3-23*01 CDR1 and CDR2 regions in NTS and TS, and a six consecutive CCCCCC site on the TS, are indicated by arrows.

Transcription occurs over the entire length of the 178 bp DNA substrate with ∼9–16% template usage (Figure 1B). Full-length RNA synthesis (198 nt RNA transcripts) by Pol II (1.4%) is stimulated by DSIF alone (8.9%, Figure 1B, C, lane 5), by AID alone (13.8%, Figure 1B, C, lane 4) or by both together (14.3%, Figure 1B, C, lane 6). Short-length Pol II transcriptional pause sites located ∼1–12 nt from the end of the scaffold bubble to a run of six consecutive Cs are observed with and without DSIF (∼52% to 65% of extended transcripts, Figure 1B, C). Intermediate-length pause sites at approximately 30 to 50 nt appear to be reduced, and longer pause sites at 100 nt appear to be eliminated in the presence of DSIF or DSIF + AID with concomitant enhancement of the full-length transcript (Figure 1B, C). An important facet of this transcriptional elongation system (Figure 1A) is that since re-initiation of transcription cannot occur from the scaffold bubble, the spectra and spatial distributions of AID-catalyzed dC deaminations, observed as NTS and TS C→T mutations in IgV (Figure 2), occur during a single round of transcription.

### AID acts on both NTS and TS DNA about equally during transcription with Pol II ± DSIF

During transcription by Pol II, AID-catalyzed C → T IgV NTS and TS mutations occur at comparable frequencies on both DNA strands (NTS = 30.2 × 10^−5^ ± 7.4) and (TS = 46.8 × 10^−5^ ± 8.0) (Table 1). The mutations are distributed across the entire IgV DNA (Figure 2). Mutations are elevated in WRC motifs, and in overlapping WGCW hot motifs on opposing strands, in CDR1 and CDR2 domains (Figure 2A, ‘red’ bars, NTS upper panel, TS lower panel), in agreement with AID deamination motif preferences measured *in vivo* (49,50). The average mutation rate per dC site is elevated on the TS (46.8 × 10^−5^) relative to the NTS (30.2 × 10^−5^), although this average difference is not statistically significant (*P* = 0.098), and can be attributed principally to a cluster of mutations occurring in a region containing 6 consecutive C residues on the TS (Figures 1B and 2A). This C-rich region is located adjacent to closely spaced transcriptional pause sites (6–11 nt) near the end of the transcription-initiation scaffold bubble (Figure 1B) that could allow more time for AID to act within or perhaps proximal to a retarded or stalled transcription bubble.

The presence of DSIF leads to a >2-fold reduction of TS mutations in the C-rich region (Figure 2B), as well as a 1.4-fold decrease in the average TS mutation rate and a significant 1.8-fold increase (*P* < 0.05) in the average NTS mutation rate over the entire IgV region (Table 1). Notably, the WRC hot motif preferences are retained during transcription in the presence of DSIF (Figure 2B). Overall, Pol II dependent AID-catalyzed NTS and TS mutation spectra are similar in the absence and the presence of DSIF, with Pearson correlation coefficients being 0.99 and 0.95 for NTS and TS spectra in Figure 2A and B, respectively. However, unlike CSR, which requires the Spt5 subunit of DSIF (32), SHM in B-cells does not require the presence of DSIF (51). Our *in vitro* results, showing that DSIF does not alter Pol II-dependent AID-catalyzed deamination spatial preferences (Figure 2) and has only a moderate effect on AID-catalyzed mutation rates on the TS (1.4-fold decrease) and NTS (1.8-fold increase) (Table 1), are largely consistent with the DSIF knockdown data in Ramos B-cells (51).

When AID alone is incubated with the scaffold bubble substrate in the absence of Pol II, AID mutations are concentrated almost exclusively within the transcription scaffold bubble and located at two WRC motifs, one motif on each DNA strand (Table 2). Although neither Pol II nor DSIF exerts a significant effect on AID catalytic activity on ssDNA (Supplemental Figure S3), the presence of Pol II strongly stimulates mutations in the scaffold transcription bubble, 11-fold on the NTS and 6-fold on the TS. There is an additional 1.5- to 2-fold stimulation of mutations in the scaffold bubble on both strands in the presence of DSIF (Table 2). These data indicate that, in this simplified Pol II transcription elongation system, AID can be present along with Pol II, or perhaps recruited by Pol II to the scaffold bubble prior to transcription elongation, and that its mutator activity when acting within the scaffold bubble is influenced significantly by Pol II and moderately by DSIF.

**Table 2.** Effect of Pol II and DSIF on AID-catalyzed deamination at 5'WRC motifs within the scaffold bubble

Strand	Mutation rate^a^ (× 10^−5^)
NTS + AID	0.6 ± 0.1
NTS + Pol II + AID	6.4 ± 2.4
NTS + Pol II + AID + DSIF	12.0 ± 0.1
TS + AID	1.4 ± 0.2
TS + Pol II + AID	9.1 ± 1.4
TS + Pol II + AID + DSIF	12.5 ± 1.1

^a^The values represent average ± standard deviation from two to three independent experiments.

We’ve determined that the spatial mutational profile for the subset of clones that have mutations both in the scaffold bubble and in the IgV body (Supplemental Figure S4) is virtually identical to another subset of clones with one IgV mutation (Supplemental Figure S5). Both subsets containing a single mutation in IgV have essentially the same mutational profile as observed for the full ensemble of singly and multiply mutated IgV clones (Figure 2). These data suggest that AID typically loads with Pol II, in either the presence or the absence of DSIF, in the scaffold bubble and stays bound during IgV transcription. Were AID to bind after transcription had already begun, then one would expect to observe an increased frequency of mutations skewed away from the scaffold bubble. Alternatively, an increased frequency of mutations located closer into the scaffold bubble would be seen if AID were to dissociate during transcription. Representative NTS and TS clones are shown for the purpose of illustrating different classes of mutational patterns that occur downstream from a mutated scaffold bubble (Supplemental Figure S6): (i) an individual mutation near the end of IgV (upper NTS and TS clones); (ii) two well-separated individual mutations (middle NTS and TS clones); (iii) individual mutations and mutational clusters (bottom NTS and TS clones). These latter clones containing mutational clusters suggest that Pol II pauses several times and then resumes synthesis. During transcriptional pausing AID appears to be able to catalyze from two to four mutations that cluster within a 15 nt window.

### AID acts almost exclusively on NTS DNA during transcription with T7 Pol

**Figure 3.**
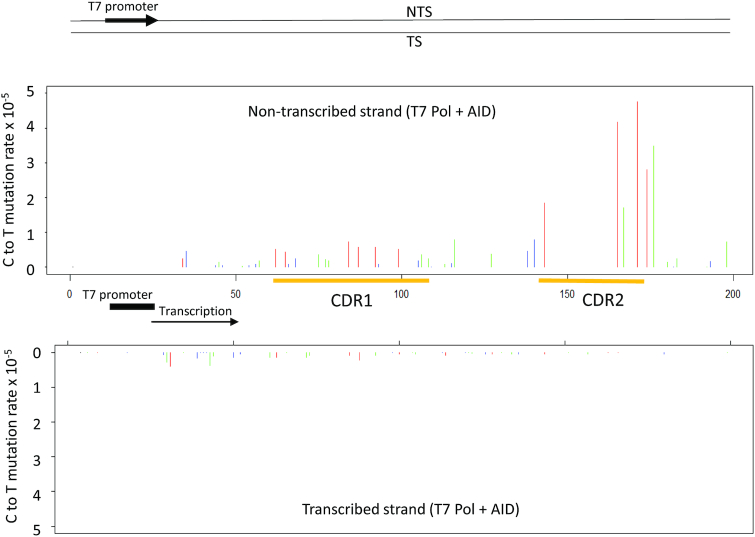
AID-catalyzed dC NTS and TS deamination spectra on IGHV3-23*01 during transcription with T7 Pol. An IGHV3-23*01 dsDNA substrate, containing a T7 promoter, was transcribed in the presence of AID and analyzed by MDS. The C → T mutation rate is shown at each dC site in the IgV target sequence (5′WRC hot-motifs, *red* bars, 5′SYC cold-motifs, *blue* bars; all other motifs containing a C site, *green* bars).

In contrast to comparable levels of NTS and TS mutations during transcription with Pol II in the presence or absence of DSIF (Figure 2A, B), mutations are observed almost exclusively on the NTS when T7 Pol is used to transcribe IGHV3-23*01 (Figure 3). AID-catalyzed NTS mutations during T7 Pol transcription also occur preferentially in CDR1 and CDR2 of IGHV3-23*01, at WRC hot motifs and intermediate motifs (Figure 3, ‘red’ and ‘green’ bars, respectively), but with mutations reduced by ∼2-fold in CDR1. The transcription bubbles for T7 Pol and Pol II are similar in size (8–10 nt), but transcriptional elongation rates are rapid with T7 Pol (∼250 nt/s) (52) and 25-fold slower with mammalian Pol II (∼9 nt/s) (43). Eukaryotic transcription is notably inefficient, experiencing frequent pausing and Pol II backtracking (43,48,53). TIRF-FRET microscopy single molecule imaging shows that AID tracks with T7 Pol in moving and retarded transcription bubbles (40) but does not bind directly to T7 Pol (40), whereas AID binds to Pol II (30) and to the Spt5 subunit of DSIF (32). Structural studies with yeast (47) and with mammalian Pol II elongation complexes (44), with DSIF present in both studies, show Spt5 binding to the NTS and TS at the upstream edge of a transcription bubble.

### Analysis of spatial mutational distributions for individual IgV DNA molecules

Our measurement for the spatial distribution of mutations on individual IgV molecules provides important new insights into scanning and catalysis during a single round of transcription. When acting in the absence of transcriptional constraints, AID scans ssDNA bidirectionally in short slides and hops (46,54). Catalysis occurs processively, with AID remaining bound to one substrate molecule with a median lifetime of ∼3 min (46) that allows each ssDNA substrate molecule to accumulate combinations of single mutations and clusters of multiple mutations (11,46,54,55). In contrast, when AID acts in a much more constrained manner during Pol II-mediated transcription, ∼67% of transcribed clones contain 1 NTS or TS mutation either in the presence or absence of DSIF (Supplemental Figure S7). The remaining multiply mutated clones (∼33%) contain two to seven mutations, with the number of mutations per clone showing an approximate exponential decrease (Supplemental Figure S7). A comparison of the spatial distribution of mutations for all clones (Figure 2) with clones having one mutation (Supplemental Figure S5) shows a reduced (∼ 4-fold) mutation frequency for singly mutated clones, but with no apparent change in the spatial mutation profiles. Therefore, the same sites that are mutated in clones with one mutation are also mutated repeatedly in multiply mutated clones.

An analysis of the distribution of mutations on the multiply mutated clones can be used to determine if AID retains the ability to perform processive catalysis in the presence of motional constraints imposed during transcription. Defining mutational clusters as the occurrence of at least two mutations within a window containing N nucleotides, then for clones containing two to five mutations, 34% of the clusters occurring on the NTS in the presence or absence of DSIF lie within a window of 1–10 nt (Supplemental Table S1). On the TS, the fraction of clusters lying within a 1–10 nt window is ∼27%. Therefore, sizable numbers of mutational clusters occur on the NTS and TS within a range of ∼10 nt, which corresponds to the estimated size of a Pol II transcription bubble (44,47).

**Figure 4.**
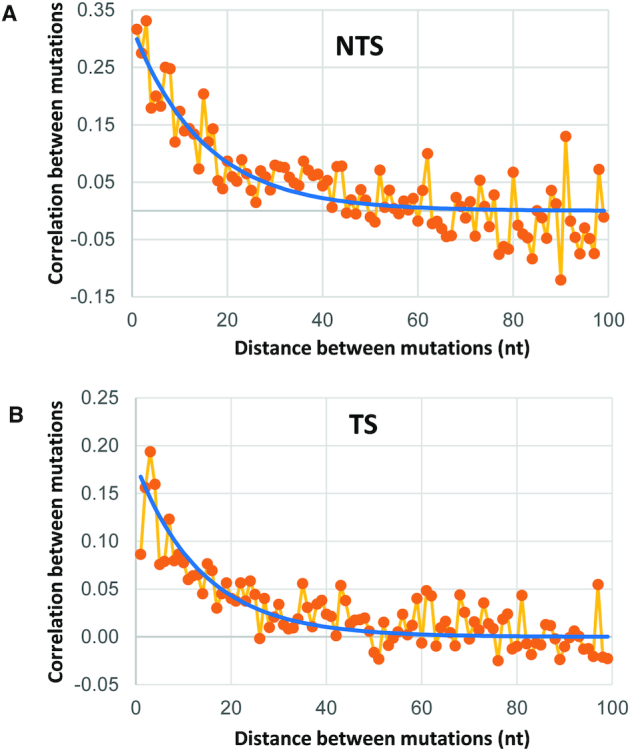
Analysis of mutation correlation in clones with two or more mutations on NTS (**A**) and TS (**B**) strands. Plots show correlation between mutations as a function of sequence separation between them on clones with more than one mutation. Exponential models (blue lines) reveal approximate correlation lengths of 15 for NTS and 14 for TS.

A quantitative measure of AID processivity can be expressed in terms of a correlation length. The correlation length is obtained by calculating the covariance, *c*(*i*,*j*) between mutations occurring at any two sites *i* and *j* located on a single DNA clone. The statistical analysis is given in Methods. A value of *c*(*i*,*j*) > 0 indicates that two mutations are positively correlated. We have calculated correlation lengths for AID of 15 nt on the NTS and 14 nt on the TS (Figure 4). An ∼3-fold reduction in correlation length compared to unconstrained scanning and catalysis on ssDNA (correlation length = ∼50 nt) (56) is almost certainly caused by a restricted distance over which AID can scan rather than an inhibition in the deamination catalytic activity of AID during transcription. We conclude, therefore, that AID retains the ability to perform processive scanning and catalysis when restricted to act either within the narrow confines of a transcription bubble, or on a ssDNA region adjacent to the bubble.

We have illustrated mutational clusters by showing several clones in which two to five mutations are concentrated in a 10–15 nt region (Supplemental Figure S8). The entire mutational distribution for the ensemble of transcribed IgV clones is shown in Figure 2. The clustered mutations appear to distribute randomly along IgV and do not concentrate at identifiable transcriptional pause sites. However, there is a mutational cluster on the TS containing six consecutive C residues located proximal to a strong transcriptional pause site (Figures 1B and 2A). This region on the TS has the highest mutation frequency, ≥2-fold higher than any of the mutationally favored WRC hot motif sites (Figure 2A). In clones with exactly one mutation, 30% are mutated within this region. In clones with two mutations, 10% are mutated at two adjacent sites, while in clones with >2 mutations, as many three of the six C sites are mutated on a single IgV DNA (Supplemental Figure S8). Single molecule experiments have shown that yeast Pol II pausing can be relatively long (>20 s) with a backtrack length of up to 8 nt (53), which can provide sufficient space and time for AID to carry out multiple correlated deaminations over ∼15 nt along the TS and the NTS. Since a large majority of multiply mutated IgV molecules, including those containing mutational clusters, do not appear to concentrate at Pol II pause sites (Figure 1B), the TS and NTS mutation distributions along IgV may reflect the presence of pause sites that arise stochastically on individual IgV DNA molecules.

### Comparing AID-Pol II with B-Cell C→T mutational spectra in an IgV target gene

**Figure 5.**
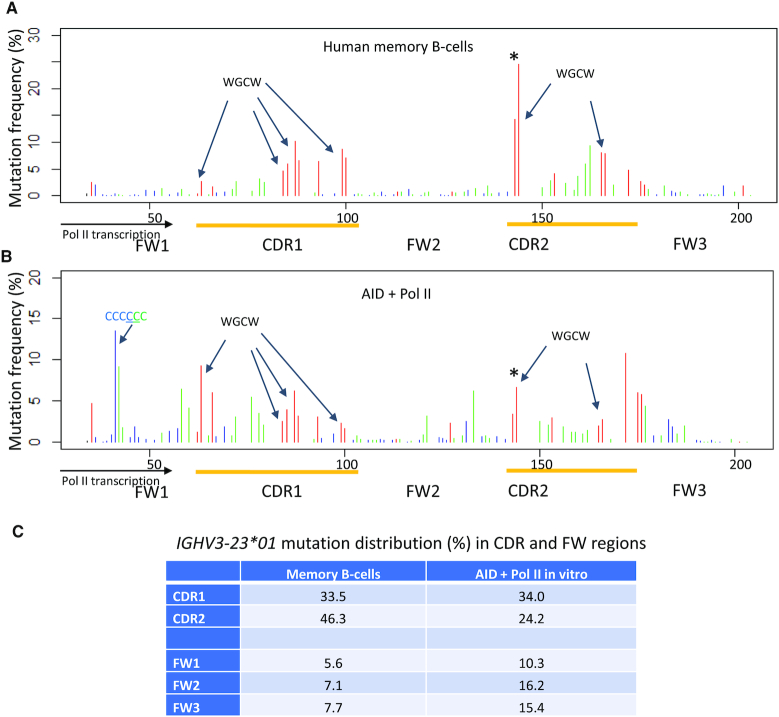
Distribution of C/G mutations from mutated non-productive human IGHV3-23*01 in memory B-cells *in vivo* (**A**) and Pol II-driven AID deamination *in vitro* (**B**). *In vivo* mutations were extracted from the memory B-cells database, and *in vitro* C:G mutations on Pol II-transcribed IGHV3-23*01 were compiled from C to T mutations on both NTS and TS strands (Figure 2A). The x-axis shows base positions within IGHV3-23*01 with designated FWs and CDR regions (yellow). The mutation frequency (%) is shown at each C or G site in the target sequence: 5′-WRC (GYW-3′) hot-motifs, *red* bars; 5′-SYC (GRS-3′) cold-motifs, *blue* bars; all other motifs contain a C or G site, *green* bars. Mutation frequency is defined as % of mutations occurring at the indicated position on TS or NTS relative to the total number of mutations found on TS or NTS. Preferred overlapping hot motifs (WGCW) in IGHV3-23*01 CDR1 and CDR2 regions in NTS and TS, and a six consecutive CCCCCC site on the TS, are indicated by arrows. An asterisk (*) above the first WGCW motif in CDR2 indicates the AGCT overlapping site that dominates the mutation spectra in memory B-cells. (**C**) Distribution of mutations (%) in CDR and FW regions of IGHV3-23*01. Mutations from six consecutive C sites were excluded for AID + Pol II *in vitro* due to frequent Pol II pausing located immediately downstream from the scaffold bubble.

We have chosen the most highly used V-region during normal immune responses, IGHV3-23*01 (36,37), to compare AID–Pol II biochemical spatial mutational profiles with human memory B-cell profiles. The portion of IGHV3-23*01 used for the *in vitro*–*in vivo* comparison contains five discrete regions: two antigen-binding complementary determining regions, CDR1 (nt 66–106); CDR2 (nt 147–177), and three Ab structural framework regions, FW1 (nt 38–65); FW2 (nt 107–146); FW3 (nt 178–203) (Supplemental Figure S1). The *in vivo* mutations were obtained from a subset of non-productive mutated IGHV3-23*01 clones, which in human circulating memory B-cells have not undergone Ag selection (49,50). *In vivo* mutations at C:G sites are presumed to result directly from NTS and TS deamination by AID, but are, however, also subjected to processing of U•G mispairs by BER and MMR (49,50). We have compared spatial mutational profiles for B-cells (Figure 5A) and AID–Pol II (Figure 5B).

The two CDRs contain large numbers of WRC and WGCW hot motifs (ten in CDR1 and six in CDR2). Mutations at hot motif sites are favored in both CDR domains for B-cells (Figure 5A, ‘*red*' bars) and for AID-Pol II (Figure 5B, ‘*red*' bars). The overlapping AGCT hot motif in CDR2 (nt 144) is the most prominently mutated site in B-cells (50) (Figure 5A, ‘*asterisk* *’). It also is mutated to a significant extent with AID – Pol II (Figure 5B, ‘*asterisk* *’), although several other CDR hot motifs are mutated to a similar extent (Figure 5B). CDR non-hot motifs are mutated at moderately high frequencies *in vivo* and *in vitro* (Figure 5A and B ‘*green*’ bars). Mutations at cold motifs (SYC) are barely detectable (Figure 5A, B, ‘*blue*’ bars).

The three FWs contain mostly non-hot and cold motifs, along with a single WRC motif in FW1, two in FW2, and three in FW3. Mutations are strongly suppressed in each FW domain in B-cells (Figure 5A). In contrast, FW mutations occur more prominently with AID – Pol II (Figure 5B). The most frequent FW mutations occur at two nearby sites in a run of six consecutive C residues located on the TS (Figure 1B). This strongly mutated FW1 region lies within 15 nt of a strong transcriptional pause site (Figure 1B), which would likely afford ample time for AID to act. This transcriptional pause site is almost certainly absent in B-cells *in vivo*. The pause site is reduced in intensity with Pol II + DSIF and is reduced further with Pol II + DSIF + AID (Figure 1B). The mutation frequencies on the TS are reduced concomitantly in the presence of Pol II + DSIF + AID (Figure 2B, bottom) compared to TS mutations with Pol II + AID (Figure 2A, bottom), which is consistent with the reduction in pausing.

To examine the relationship between Pol II pausing or termination and AID-catalyzed deamination, we have performed RNA-seq analysis of a Pol II transcription reaction in the presence of AID. The distribution of RNA transcripts having 3′-ends terminated at template positions at the 5′ side of IgV covering a portion of FW1 and the entire CDR1 and FW2 regions is shown in Supplemental Figure S9A. Since Pol II produces numerous short transcripts in the FW1 region just downstream from the scaffold bubble (Figure 1B, C, Supplementary Figure S9A), we have focused on comparing paused or terminated transcripts in CDR1 and FW2 regions. Pol II appears to either pause or terminate at C:G template sites (‘*colored*’ bars) and A:T template sites (‘*gray*’ bars). The average number of RNA transcripts with 3′-ends in CDR1 (2830 ‘counts’ per site) is significantly higher than the average number of transcripts for FW2 (487 ‘counts’ per site) (*P* = 0.002, two-tailed Student's *t*-test). Thus, a higher density of Pol II paused or terminated transcripts in CDR1 (Supplemental Figure S9A) appears to correlate reasonably well with higher AID-induced mutations (Supplemental Figure S9B), compared to a lower density of paused or terminated transcripts and lower mutation rates in FW2.

The main feature of the memory B-cell *in vivo* (Figure 5A) and AID – Pol II biochemical (Figure 5B) comparison is that AID-initiated mutations exhibit, for the most part, similar spatial mutation profiles at C:G sites. Correlation analysis of the memory B-cell and AID-Pol II *in vitro* IgV spectra revealed a moderate Pearson correlation coefficient, 0.33 in the absence of DSIF and 0.41 in the presence of DSIF. Differences in the two mutational profiles, which are more pronounced in FWs (Figure 5C), lends emphasis to the to the well-known point that downstream processing of U•G mispairs by BER and MMR significantly alter the spatial distribution of IgV mutations. Reduced mutational levels in B-cells in the three FW regions are important in maintaining the overall Ab structure and function.

## DISCUSSION

A primary motivation for investigating AID catalytic properties during transcription elongation by human Pol II was to establish whether or not dC deamination occurs on both NTS and TS DNA in the absence of additional transcription proteins or AID transcriptional targeting factors. *In vivo*, SHM occurs at C sites at about equal frequencies on both DNA strands, favoring WRC and WGCW motifs mainly in IgV CDR regions. If comparable spatial distributions of deaminations were also found to occur on both strands *in vitro*, then the inclusion of AID in a mammalian transcription system that includes an IgV and IgS promoter and transcription initiation factors would offer a first step toward the reconstitution of SHM and CSR with purified enzymes. Our transcription elongation system, which includes AID and human Pol II in the presence or absence of DSIF, has an important technical feature; since transcriptional re-initiation cannot occur, the spatial mutational patterns on individual clones, and for mutational spectra for the ensemble of clones, reflect transcription-dependent dC deamination by AID that occurs during a single transcriptional cycle. Our biochemical data show that the presence of AID and Pol II are sufficient to generate transcription-dependent dC deamination about equally on both DNA strands. In accord with *in vivo* data, mutations are favored in WRC and WGCW motifs on both NTS and TS in the two CDR regions of IGHV3-23*01. A comparison of biochemical data with B-cell data for the same IgV target motif show good qualitative agreement for spectral mutational profiles for the densely mutated CDRs and reasonable, albeit somewhat less agreement in the more sparsely mutated FW regions.

In B-cells, AID targets IgV and IgS regions with high efficiency along with multiple off-target loci (57,58) at greatly reduced efficiencies. Genome-wide ChIP coupled with deep sequencing measurements have revealed that AID appears to associate with sizable numbers of genes in activated B-cells, in excess of ∼5900 (31). These observations suggest that the vast majority of genes that, in principle, can interact with AID do not undergo measurable mutation. Several factors, such as Pol II, DSIF, PAF1C and an RNA processing exosome, have been implicated in AID genomic localization and attendant mutagenic activity. The Spt5 subunit of DSIF exhibits a peak genomic distribution that correlates strongly with genomic localization of AID (31,32). Reduction of Spt5 levels via knockdowns in CH12 cells show significantly reduced CSR (59), suggesting that Spt5 is required for CSR (32,59). In contrast, a reduction in Spt5 levels in Ramos B-cells leads to a slight increase in IgV SHM (51). The differential effects of Spt5 for SHM and CSR could reflect different ways in which AID gains access to IgV and IgS regions during Pol II transcription. It has been suggested that stalled or premature transcription termination provides ssDNA substrates for AID during SHM, whereas R-loop structure formation in IgS undergoing transcription serves as the major source of ssDNA during CSR (51). Our *in vitro* data show that IGHV3*23 NTS mutation rates are increased by <2-fold in the presence of DSIF (Table 1 and Figure 2), which is consistent with a limited role for DSIF during SHM (51). Nonetheless, the possibility remains that in concert with other factors in vivo (e.g. PAF1C), DSIF can affect mutation frequency.

Since AID cofactors, Pol II, Spt5 and PAF1C, are distributed throughout the genome, a central question of how AID targets Ig genes with high specificity in B-cells remains unresolved. AID interactions with Pol II transcription machinery and perhaps other Ig loci-specific cofactors are likely to be needed for SHM and CSR (59). Alternatively, however, there may also be factors that might act to prevent AID binding to ssDNA during the transcription of non-IgV or IgS regions. Our *in vitro* data show that AID is able to deaminate NTS and TS DNA using just a minimal Pol II transcription elongation system in the absence of transcription factors. Two other poorly understood observations are that SHM is initiated at ∼185 bases downstream of the promoter and that mutations are terminated ∼1.5–2 kb further downstream, stopping prior to the C region (60–62). Regarding the spatially delayed appearance of mutations, it has been speculated that AID might be excluded from a region extending from the promoter to 150 bp downstream because of a high density of TFIIH and transcription initiation factors bound to promoters (51). Currently there are no biochemical data to explain how SHM occurs exclusively on transcribed IgV DNA, while absent on a contiguous IgC region that is also undergoing transcription.

**Figure 6.**
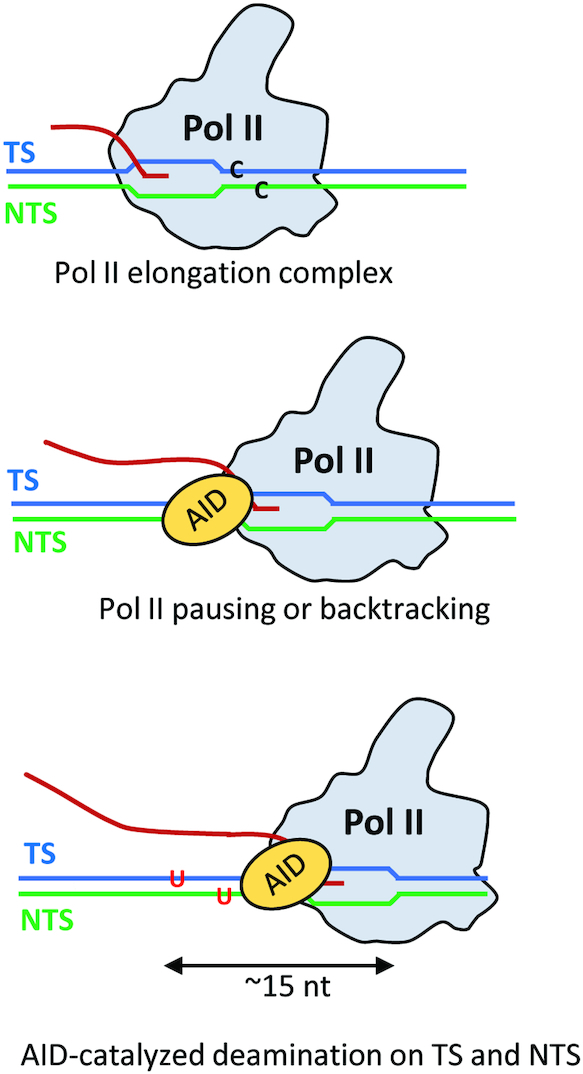
A model for AID putative access to the NTS and TS during Pol II transcription. Sketch of how AID might interact with dC residues on both the TS or NTS to convert C → U. Pol II has been observed to pause and to backtrack during transcription elongation. We propose that paused or backtracked Pol II interacts with AID at the upstream edge of the transcription bubble where the TS and NTS strands exit the polymerase, as inferred from structural studies of Pol II elongation complexes. In this model, AID can interact with dC residues on both the TS or NTS to convert C → U. Structural and single-molecule resolution transcriptional data suggest that AID could have access to about a 15 nt region of transient ssDNA, corresponding to a stalled transcription bubble (∼10 nt) and perhaps an additional region of ssDNA resulting from a backtracked Pol II (∼5 nt).

How might AID gain access to both DNA strands during Pol II transcription? X-ray crystallographic and CryoEM data with Pol II show a densely packed enclosure surrounding the transcription bubble, seemingly affording AID little if any access to either the NTS or TS (25,29,44,47). However, the structures indicate nascent RNA transcript displacement from the bubble and passage through the RNA exit channel as the TS and NTS strands exit Pol II (25,29,47). Since AID interacts with Pol II, we speculate that AID may bind to Pol II, perhaps in conjunction with DSIF, at the upstream edge of the transcription bubble where TS and NTS strands are extruded from the bubble (Figure 6). An example for this kind of interaction with the Pol II transcription bubble has been recently reported for the transcription-coupled repair protein Rad26 (63). A cryoEM structure of a yeast Pol II-Rad26 elongation complex indicates that Rad26, acting as a ssDNA translocase, pulls the TS strand away from stalled or paused Pol II and thereby promotes Pol II forward translocation (63).

Despite having important structural similarities with multi-subunit RNA Pol structures, single subunit T7 RNA Pol does not interact directly with AID (40). The T7 elongation complex also has much shorter DNA and RNA entry and exit channels compared to multi-subunit polymerases, which may contribute to a much faster transcription rate for T7 Pol (200–300 nt/s (64)) compared to Pol II (∼9 nt/s (43)). A reduction in rNTP substrate concentrations during T7 RNA pol transcription results in an increase in mutational clusters, suggesting that rapid transcription reduces the ability of AID to gain access to ssDNA formed transiently in rapidly translocating transcription bubbles (65). Thus, each of these factors: the absence of a direct interaction of AID with T7 RNAP (40), rapid transcription, and infrequent pausing may constrain the action of AID to the NTS (Figure 3) (11–13).

It has been proposed that transcriptional pausing, backtracking, and perhaps even premature termination (32,51,65) may be needed to provide sufficient time for the catalytically slow AID (∼0.03/s for favored WRC motifs (46,54)) to generate SHM efficiently during transcription of IgV. Our RNA-seq and mutational data showing a higher density of Pol II pausing or termination in CDR1, which coincide with higher AID-induced mutations, compared to a lower density of Pol II pausing or termination and lower mutations in the FW2 region (Supplemental Figure S9), appear to be consistent with the above hypothesis. It is important to note that there is no site-to- site correlation between Pol II paused or terminated transcripts and AID-induced mutations favored at WRC motifs. For example, only two out of four WGCW sites in CDR1 showed high frequencies of paused or terminated transcripts and several sites in FW2 and CDR2 showed high mutation rates with relatively low amounts of paused or terminated transcripts (Supplemental Figure S9A and B).

AID scans ssDNA randomly in bidirectional slides and short hops with no apparent directional catalytic bias (46,54). Presumably, therefore, AID can track along with Pol II and act with similar efficiencies on exposed NTS and TS DNA while scanning ssDNA within a stalled or backtracked transcription bubble. Our experiment detects dC deaminations on both strands in a large ensemble of transcribed DNA molecules, but it does not detect NTS and TS deaminations occurring on the same dsDNA molecule. Crystal structures of monomeric AID variants suggest that one AID molecule is likely to accommodate a single ssDNA substrate (66,67). If that proves to be the case, then it will be important to determine how a single bound AID can switch between TS and NTS ssDNA regions during transcription of IgV DNA. Alternatively, it is possible that an AID dimer is needed to access the two strands. A dimeric form of AID appears to be required during CSR (68) in a transcriptional process that typically involves the formation of sizable D-loops in Ig switch regions in contrast to transcription of IgV. Structural imaging of Pol II with AID on model transcription bubble substrates could reveal AID stoichiometry and TS and NTS interaction sites during SHM.

The influence of transcription on AID deamination mechanisms can be inferred by analyzing spatial mutation distribution profiles obtained by next-generation sequencing of large numbers of singly and multiply mutated independent DNA clones. About 67% of the clones have one mutation and 33% have between two and seven mutations (Supplemental Figure S7). The spatial profiles for singly and multiply mutated clones are virtually indistinguishable and contain mutations distributed throughout the entire 178 bp IGHV3-23*01 DNA. When acting on an ssDNA substrate, AID scans along the backbone bidirectionally and deaminates dC target residues processively (11). Although catalysis is processive, it is inefficient, even in favored WRC motifs, which generates widely varying patterns of isolated and clustered mutations (11,46,54). These same properties of AID that are observed during unconstrained scanning of ssDNA also carry over during much more constrained scanning during transcription of dsDNA. During transcription, multiply mutated clones can contain numerous mutational clusters that have at least two mutations situated within a 10 nt window (Supplemental Table S1), suggesting the presence of processive catalysis during transcription. We have calculated a correlation length of ∼15 nt by computing the mutational covariance for the ensemble of multiply mutated clones (Figure 4). A 15 nt correlation length implies that mutations that occur within the approximate distance of a transcription bubble (or backtracked Pol II) are very likely to have been generated by the processive catalytic action of a single AID molecule. For comparison, a 50 nt correlation length has been observed for AID scanning unconstrained on ssDNA (56). Therefore, processive catalysis appears to be retained during transcription with Pol II, but with a reduced correlation length likely caused by spatial constraints imposed by transcription. In contrast to *in vitro* data showing correlated mutations at G:C sites that fall within a distance of ≤ 15 nt (Figure 4), human memory B-cell mutations (Figure 5A) are not correlated when subjected to the same analysis. Previous studies of clustered mutations in mouse B-cells also showed that they are essentially uncorrelated (69). It has been suggested that the presence of nucleosomes during IgV transcription, and the elimination of U•G mispairs by BER, and MMR, are responsible for suppressing the processive signature of AID *in vivo* (69).

This ‘simplest’ representation of a mammalian transcription elongation system, which includes just AID and Pol II in the presence or absence of DSIF, appears to share basic features in common with B-cell mutational spectra. Based on these results, it would now be advantageous to expand this transcriptional elongation system by including a Pol II promoter and transcription initiation factors (70,71). The use of properly initiating transcription systems, which include an IgV (or IgS) promoter, transcription initiation proteins and additional transcription elongation factors such as TFIIS/SII and PAF1C will allow for a more complete *in vitro* versus *in vivo* comparison of AID-initiated mutation spectra. For example, it is possible that the observed differences between the *in vitro* and B-cell spatial mutation distributions are wholly or partially caused by the absence of transcription factors, such as anti-pausing factor TFIIS/SII, in our ‘minimalistic transcription elongation’ system. A related consideration is the extent to which chromatin structure, as exists on Ig genes *in vivo*, modulates Pol II-dependent AID function. Given our development of biochemically defined, recombinant chromatin-templated Pol II transcription systems that mediate both accurate transcription initiation and subsequent TFIIS- and PAF1C-dependent elongation through an array of nucleosomes (72,73), it should also be possible to investigate this issue. Currently, it is not known how AID is targeted to transcribed IgV DNA, nor is it known how AID is prevented from targeting transcribed Ig constant regions. Expanded versions of our initial *in vitro* transcription system have the potential to identify and characterize AID targeting and anti-targeting factors, with a broader goal of reconstituting SHM and CSR using purified proteins.

## Supplementary Material

gkz821_Supplemental_File
