# Supplementary material for: AID–RNA polymerase II transcription-dependent deamination of IgV DNA
Source: Nucleic Acids Res. 2019 Sep 30;47(20):10815–29. doi: 10.1093/nar/gkz821 (PMC6846656; doi:10.1093/nar/gkz821)
Supplement: gkz821_Supplemental_File [file gkz821_supplemental_file.pdf]

SUPPLEMENTAL DATA

AID-RNA Polymerase II Transcription-dependent Deamination of IgV DNA

Phuong Pham[1], Sohail Malik[2], Chiho Mak[3], Peter C. Calabrese[1], Robert G. Roeder[2], and Myron F. Goodman[1,3]
[1] *Department of Biological Sciences, University of Southern California, Los Angeles, CA 90089*
[2] *Laboratory of Biochemistry and Molecular Biology, The Rockefeller University, New York, NY 10065*
[3] *Department of Chemistry, University of Southern California, Los Angeles, CA 90089*

Supplemental Figure S1. The cloned NTS and TS sequences of *IGHV3-23*01*

Supplemental Figure S2. SDS-PAGE analyses of purified Pol II and DSIF

Supplemental Figure S3. DSIF and Pol II exert no significant effect on AID activity on ssDNA.

Supplemental Figure S4. Deamination profile of mutated clones containing mutations in both “scaffold bubble” and IgV regions

Supplemental Figure S5. Deamination profile of AID composed from clones with only 1 mutation on the NTS and TS strand during transcription by human Pol II ± DSIF.

Supplemental Figure S6. Representative NTS and TS clones with mutations in both the scaffold bubble and IgV regions

Supplemental Figure S7. Distribution of clones with mutations in *IGHV3-23*01* NTS and TS strands

Supplemental Figure S8. Representative NTS and TS clones with 2 - 5 mutations and TS clones with more than 5 mutations

Supplemental Figure S9. Comparison of IgV Pol II pausing sites and AID-induced mutation distribution

Supplemental Table S1. Mutation cluster analysis for clones with 2 or more mutations

Supplemental Table S2. A list of DNA primers for Illumina sequencing library construction by MDS methods

Cloned NTS sequence (5'-3'):

CTGGCAGTAC TAGT**AAACTA GTATTGA**AAG TAAGCCTGGG GGGTCCCTGA GACTCTCCTG TGCAGCCTCT

CDR1

GGATTCACCT TTAGCAGCTA TGCCATGAGC TGGGTCCGCC AGGCTCCAGG GAAGGGGCTG GAGTGGGTCT

CDR2

CAGCTATTAG TGGTAGTGGT GGTAGCACAT ACTACGCAGA CTCCGTGAAG GGCCGGTTCA CAG

Cloned TS sequence (5'-3'):

CDR2

CTGTGAACCG GCCCTTCACG GAGTCTGCGT AGTATGTGCT ACCACCACTA CCACTAATAG CTGAGACCCA

CDR1

CTCCAGCCCC TTCCCTGGAG CCTGGCGGAC CCAGCTCATG GCATAGCTGC TAAAGGTGAA TCCAGAGGCT

GCACAGGAGA GTCTCAGGGA CCCCCCAGGC TTACTT**AAGC CTGGTCAT**TA CTAGTACTGC CAG

Supplemental Figure S1. **The cloned NTS and TS sequences of *IGHV3-23*01*.** Gray shaded area in NTS and TS are framework regions and green shaded sequences are complementarity determining CDR1 and CDR2. Bases in red font in NTS and TS represent the "scaffold bubble".

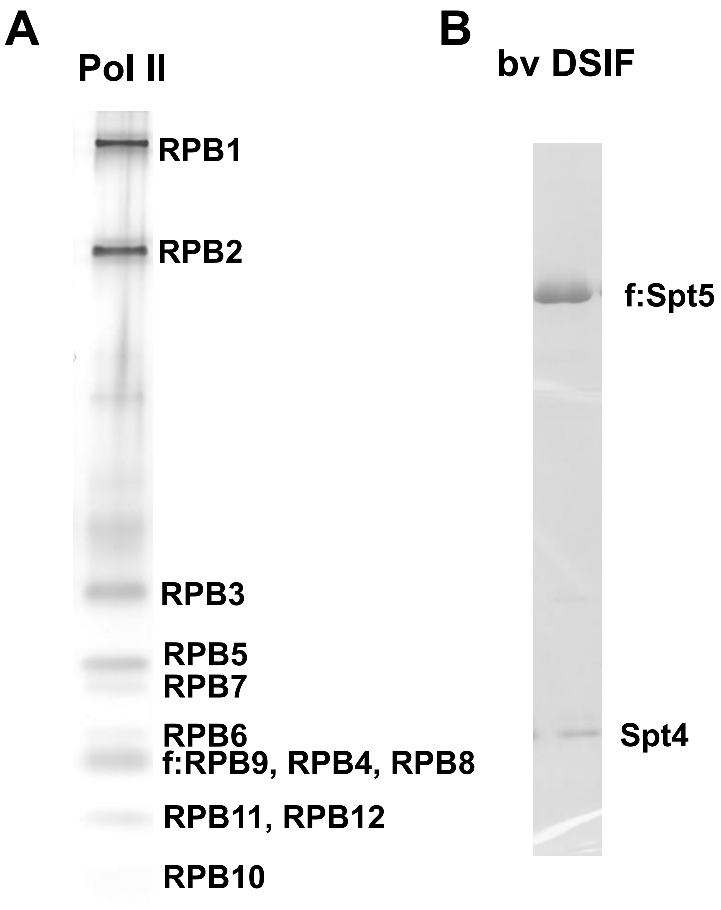

Supplemental Figure S2. **SDS-PAGE analyses of purified Pol II and DSIF. A**. The Pol II preparation was analyzed on a 4-15% gel and visualized by silver staining. **B**. The DSIF preparation was analyzed on an 11% gel and stained with Coomassie Brilliant Blue.

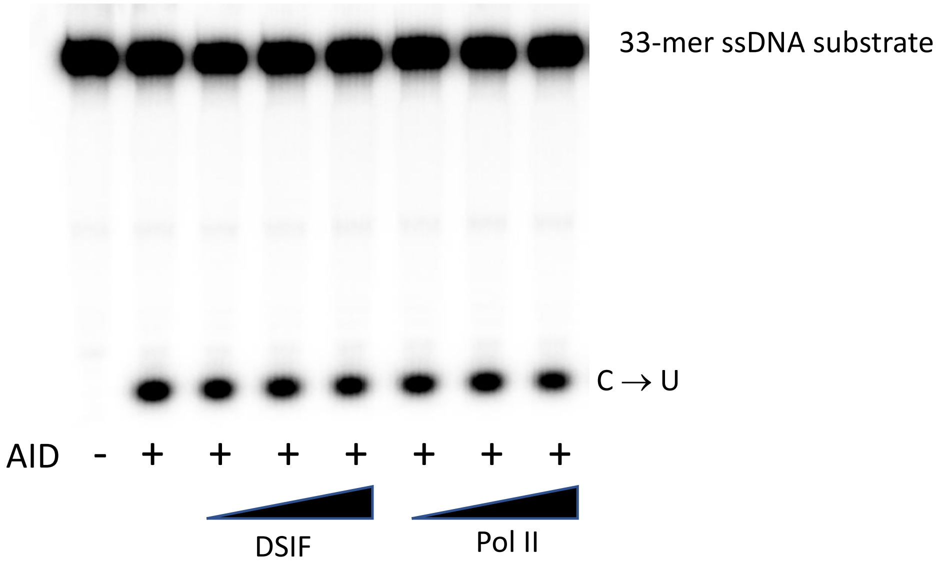

Supplemental Figure S3. DSIF and Pol II exert no significant effect on AID activity on ssDNA. ^{32}P-labeled 33-mer oligo containing a single AG<u>C</u> hot motif was used as a substrate for AID activity measurement. Reactions were carried out by incubating 2 pmol of ssDNA with 100 fmol of purified AID in 25 μl reaction buffer (10 mM HEPES-KOH, pH 8.2, 130 mM KCl, 5 mM DTT, 8% glycerol and 20 mg of BSA/ml) at 30°C for 3 min. When present, increasing amounts of DSIF (28 fmol, 142 fmol and 284 fmol) and Pol II (10 fmol, 50 fmol and 100 fmol) were added, as indicated. Reactions were terminated by twice extraction with phenol:chloroform:isoamyl alcohol (25:24:1), followed by treatments with Uracil DNA Glycosylase and hot alkali to cleave the ssDNA at the deaminated C site. Reactions products were resolved by 12% denaturing PAGE and and visualized by PhosphorImaging.

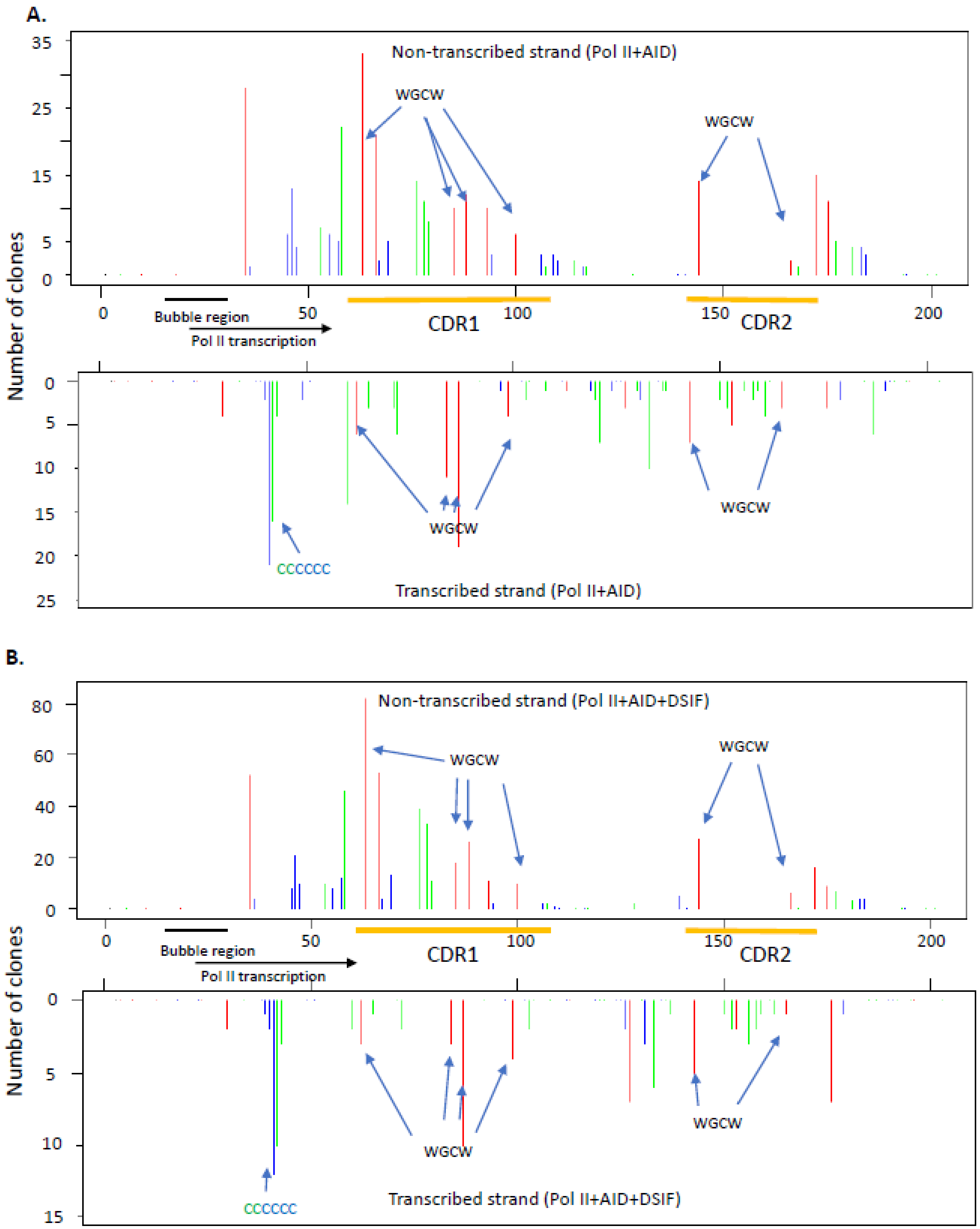

Supplemental Figure S4. Deamination profile of mutated clones containing mutations in both "scaffold bubble" and IgV regions. A, AID deamination spectra for the NTS (top) and TS (bottom) strands of Pol II-transcribed DNA in the absence of DSIF. Deaminations are detected as C → T mutations at C template sites after MDS sequencing analysis. Each colored bar represents a number of clones with a C → T mutation at the indicated position on *IGHV3-23*01* NTS and TS strands. *Red* bars identify C deaminations occurring in 5'WRC hot motifs, *blue* bar represent 5'SYC cold motifs, and *green* bars represent neither WRC nor SYC "neutral" motifs. **B**, AID deamination spectra for the NTS (top) and TS (bottom) strands of Pol II transcribed DNA in the presence of DSIF.

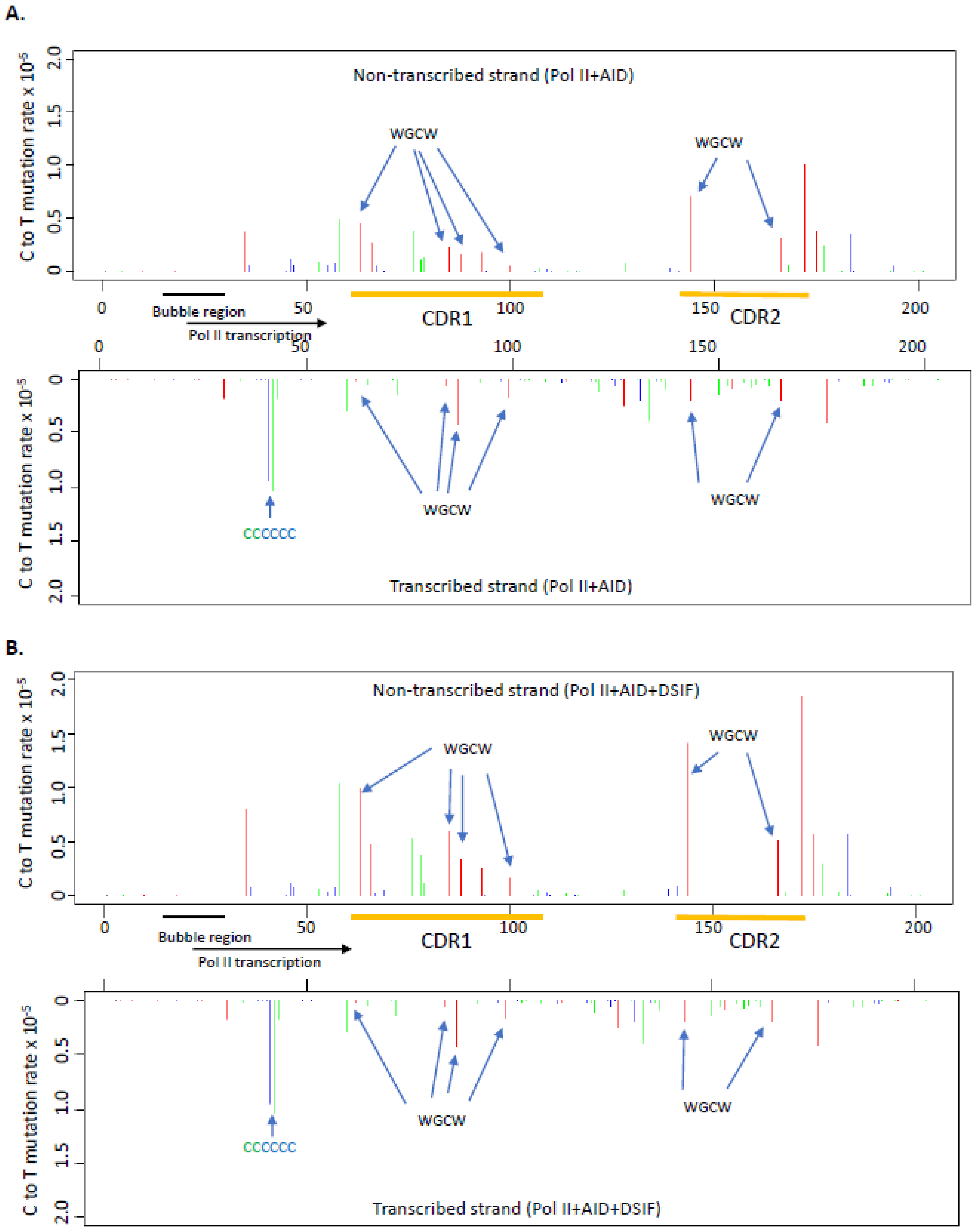

Supplemental Figure S5. Deamination profile of AID composed from clones with only 1 mutation on the NTS and TS strand during transcription by human Pol II ± DSIF. A, AID deamination spectra for the NTS (top) and TS (bottom) strands of Pol II-transcribed "scaffold bubble" DNA in the absence of DSIF. Deaminations are detected as C → T mutations at C template sites after MDS sequencing analysis. Each colored bar represents a C → T mutation rate at the indicated position on *IGHV3-23*01* NTS and TS strands. *Red* bars identify C deaminations occurring in 5'WRC hot motifs, *blue* bar represent 5'SYC cold motifs, and *green* bars represent neither WRC nor SYC "neutral" motifs. **B**, AID deamination spectra for the NTS (top) and TS (bottom) strands of Pol II- transcribed DNA in the presence of DSIF.

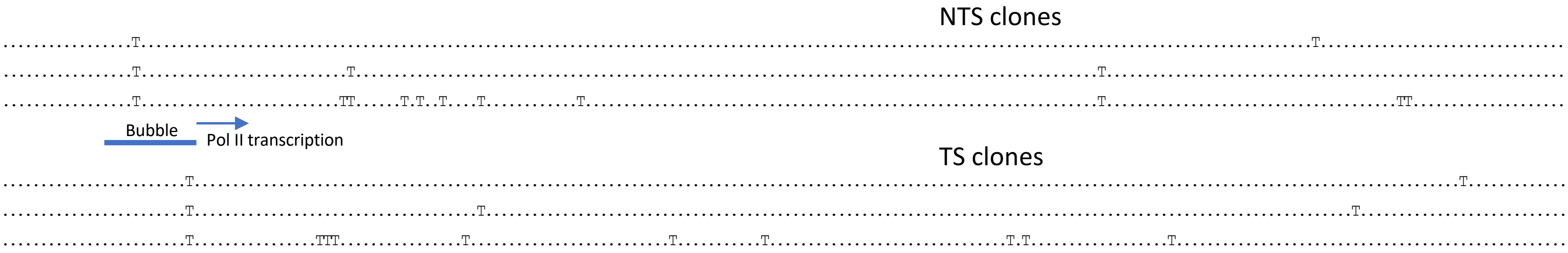

Supplemental Figure S6. **Representative NTS and TS clones with mutations in both the scaffold bubble and IgV regions.** AID-catalyzed deaminations at dC sites are converted to T, as indicated. The scaffold bubble containing an initiator RNA oligonucleotide (see Figure 1A) is indicated as “Bubble”.

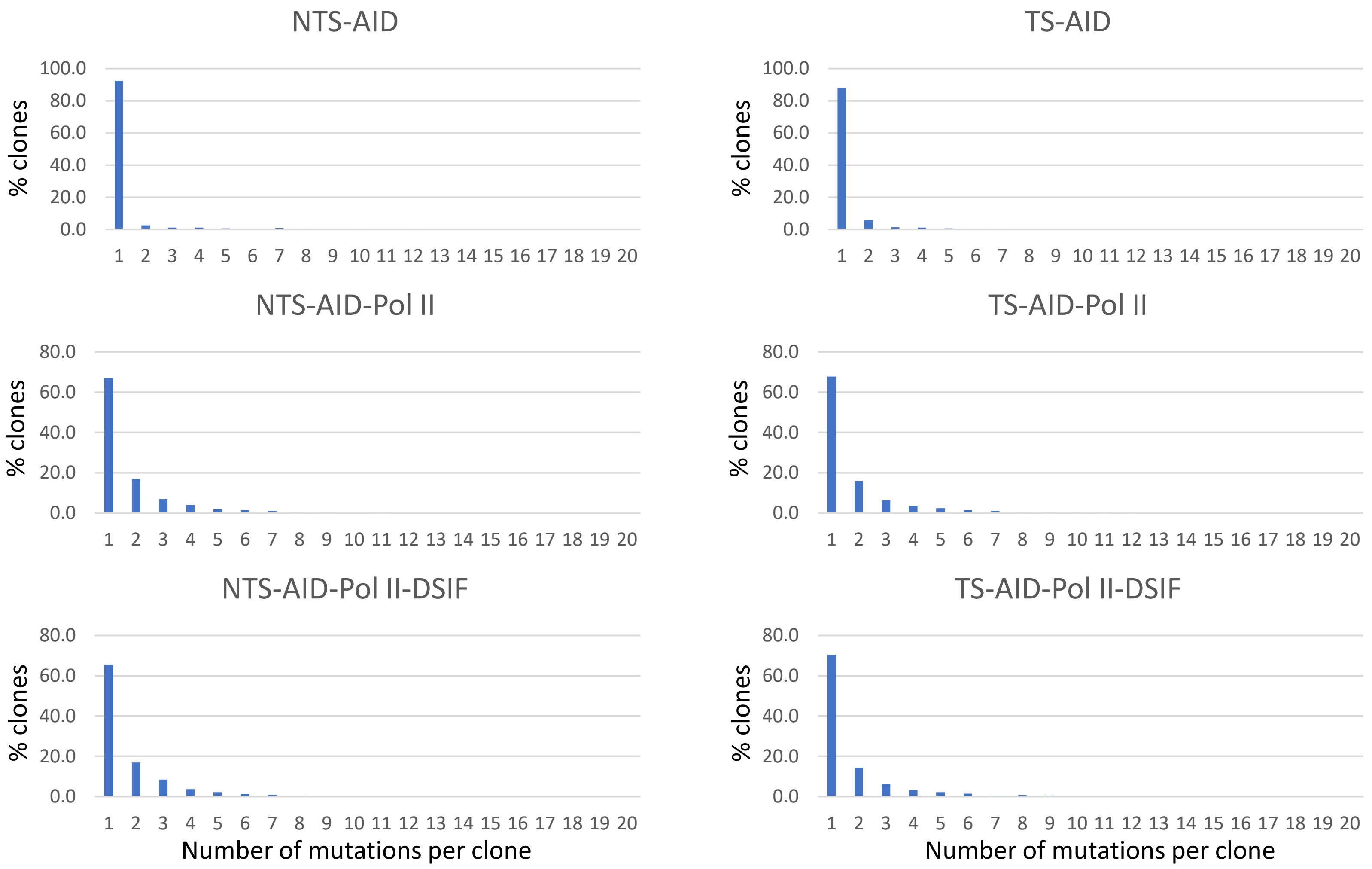

Supplemental Figure S7. **Distribution of clones with mutations in *IGHV3-23*01* NTS and TS strands.**

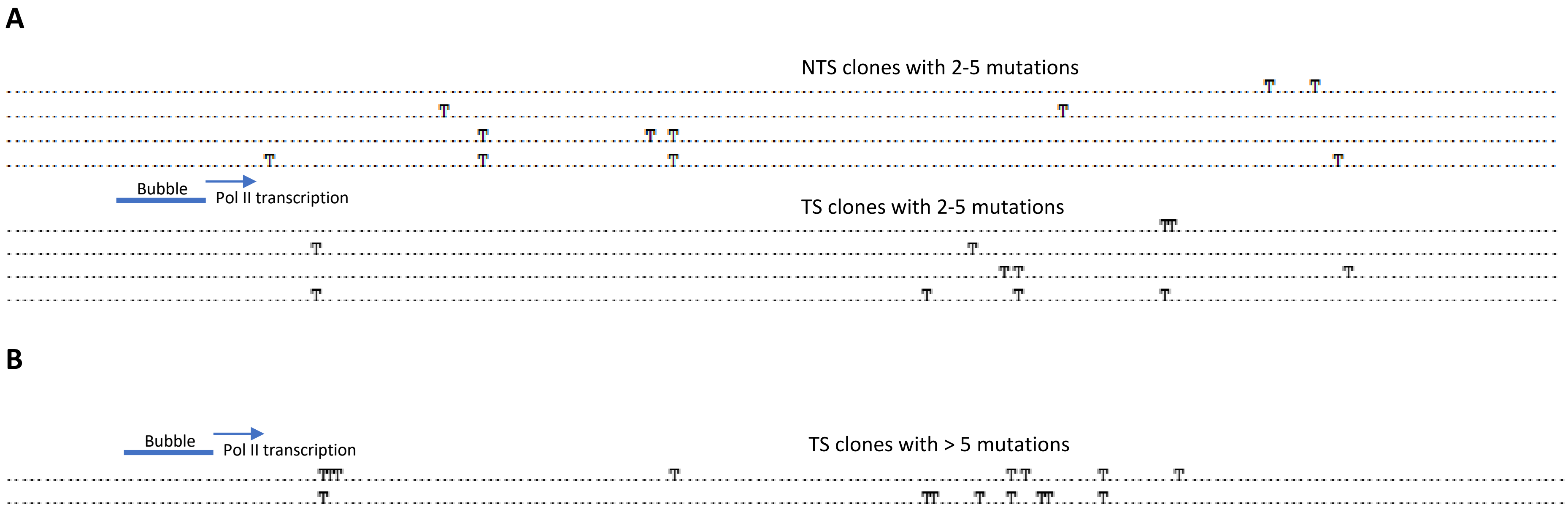

Supplemental Figure S8. **Representative NTS and TS clones with 2 - 5 mutations (A) and TS clones with more than 5 mutations (B).** The NTS clones (**A**) were selected to provide examples that illustrate the following points: 1) the presence of a single cluster (two mutations within a 10 nt window); 2) the presence of two widely spaced mutations, one near the transcription initiation site, the other toward the end of IgV transcript, showing that the two widely spaced mutations occur during one round of transcription; 3) the presence of a mutational cluster and a separated individual mutation; 4) the presence of multiple (four) separated individual mutations spanning the entire transcribed IgV region, occurring during one round of transcription. The TS clones in panel (**A**) were selected to provide examples directly comparable to the NTS clones in panel (**A**) showing that similar types of mutations occur on both NTS and TS. The TS clones (**B**) were selected to provide: (top clone) examples of multiple mutations (three) clustered in a region of six consecutive C residues at a stalled transcriptional site shown in Fig. 1B along with mutations occurring downstream, which include a cluster with two mutations along with two individual mutations that are spaced within the TS correlation length of about 14 nt; (bottom clone) a single mutation in a region of six consecutive C residues shown in Fig. 1B, along with a variety of clustered mutations occurring downstream. Each dot represents a single template base in the NTS (5′→3′) or TS (3′→5′). AID catalyzed deaminations that occur at dC sites are converted to T, as indicated. The “scaffold bubble” containing an initiator RNA oligonucleotide (see Figure 1A) is indicated as “Bubble”.

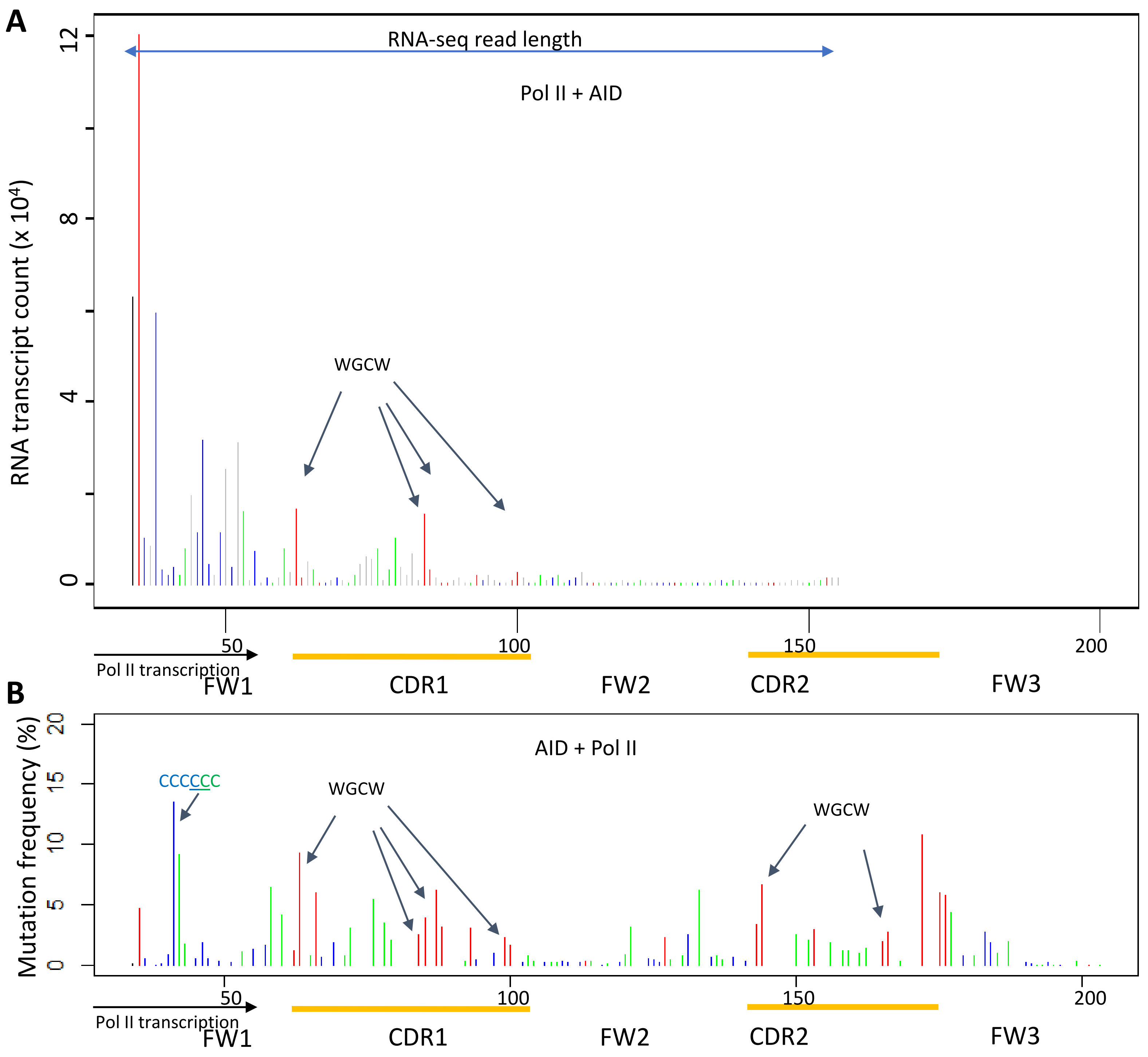

Supplemental Figure S9. Comparison of IgV Pol II pausing sites and AID-induced mutation distribution. A, RNA-seq (150 bp read length) was employed to quantify frequencies of Pol II transcription pausing or premature termination along IgV in the presence of AID. The colored bars show the distribution of IgV RNA transcripts with the 3'-end at the indicated IgV positions from 34 to 155. The transcript counts for the number of sequence reads with the 3'-end are shown at each C or G site in: 5'-WRC (GYW-3') hot-motifs, *Red* bars; 5'-SYC (GRS-3') cold-motifs, *blue* bars; all other motifs contain a C or G site, *green* bars. The transcript counts with the 3'-end at A:T sites are shown as *gray* bars. **B, Pol II-dependent AID-induced IgV deamination spectra at C:G sites for both the NTS and TS strands**. Mutation frequencies (%) are shown at each C or G site in the target sequence: 5'-WRC (GYW-3') hot-motifs, *Red* bars; 5'-SYC (GRS-3') cold-motifs, *blue* bars; all other motifs contain a C or G site, *green* bars. Mutation frequency is defined as % of mutations occurring at the indicated position on TS or NTS relative to the total number of mutations found on TS or NTS. Preferred overlapping hot motifs (WGCW) in *IGHV3-23*01* CDR1 and CDR2 regions in NTS and TS, and a six consecutive CCCCCC site on the TS, are indicated by arrows.

Supplemental Table S1. Mutation cluster analysis for clones with 2 or more mutations.

Experiment 1

Fraction of clusters with at least 2 mutations in a x-nt window

	NTS (Pol II +AID)			NTS (Pol II +AID +DSIF)			TS (Pol II +AID)			TS (Pol II +AID +DSIF)		
	2-5 mut	6-10 mut	11+ mut	2-5 mut	6-10 mut	11+ mut	2-5 mut	6-10 mut	11+ mut	2-5 mut	6-10 mut	11+ mut
5 nt window	0.15	0.11	0.10	0.17	0.10	0.08	0.19	0.14	0.07	0.17	0.13	0.17
10 nt window	0.34	0.25	0.24	0.34	0.22	0.20	0.26	0.27	0.14	0.28	0.28	0.35
15 nt window	0.46	0.37	0.36	0.44	0.35	0.31	0.35	0.38	0.20	0.39	0.41	0.45
20 nt window	0.54	0.49	0.48	0.52	0.46	0.39	0.42	0.46	0.28	0.53	0.50	0.57

Experiment 2

Fraction of clusters with at least 2 mutations in a x-nt window

	NTS (Pol II +AID)			NTS (Pol II +AID +DSIF)			TS (Pol II +AID)			TS (Pol II +AID +DSIF)		
	2-5 mut	6-10 mut	11+ mut	2-5 mut	6-10 mut	11+ mut	2-5 mut	6-10 mut	11+ mut	2-5 mut	6-10 mut	11+ mut
5 nt window	0.17	0.13	0.12	0.16	0.11	0.12	0.19	0.13	0.09	0.19	0.16	0.14
10 nt window	0.36	0.26	0.25	0.32	0.22	0.22	0.31	0.21	0.22	0.35	0.29	0.29
15 nt window	0.50	0.38	0.40	0.44	0.34	0.36	0.40	0.30	0.30	0.45	0.38	0.37
20 nt window	0.58	0.47	0.46	0.53	0.43	0.45	0.49	0.40	0.37	0.56	0.49	0.45

Supplemental Table S2. DNA primers for Illumina sequencing library construction by MDS methods.

1. DNA primers for attachment of 24 nt random barcode unique identifier (UID):

NTS strand

Ind-24-NTSC
5’CTGGAGTTCAGACGTGTGCTCTTCCGATCNNNNNNNNNNNNNNNNNNNNNNNNCTGTGAACCGGCCCTTCAC
Ind-24-NTSCT
5’CTGGAGTTCAGACGTGTGCTCTTCCGATCNNNNNNNNNNNNNNNNNNNNNNNNTCTGTGAACCGGCCCTTCAC

TS strand

Ind-24-TSC
5’CTGGAGTTCAGACGTGTGCTCTTCCGATCNNNNNNNNNNNNNNNNNNNNNNNNCTGGCAGTACTAGTAATGACCA
Ind-24-TSCT
5’CTGGAGTTCAGACGTGTGCTCTTCCGATCNNNNNNNNNNNNNNNNNNNNNNNNTCTGGCAGTACTAGTAATGACCA

N = A, C, G or T

2. DNA primers with an Illumina’s index for the linear amplification step:

NG-1
5’CAAGCAGAAGACGGCATACGAGATCGTGATGTGACTGGAGTTCAGACGT
NG-2
5’CAAGCAGAAGACGGCATACGAGATACATCGGTGACTGGAGTTCAGACGT
NG-3
5’CAAGCAGAAGACGGCATACGAGATGCCTAAGTGACTGGAGTTCAGACGT
NG-4
5’CAAGCAGAAGACGGCATACGAGATTGGTCAGTGACTGGAGTTCAGACGT
NG-5
5’CAAGCAGAAGACGGCATACGAGATCACTGTGTGACTGGAGTTCAGACGT
NG-6
5’CAAGCAGAAGACGGCATACGAGATATTGGCGTGACTGGAGTTCAGACGT
NG-7
5’CAAGCAGAAGACGGCATACGAGATGATCTGGTGACTGGAGTTCAGACGT
NG-8
5’CAAGCAGAAGACGGCATACGAGATTCAAGTGTGACTGGAGTTCAGACGT
NG-9
5’CAAGCAGAAGACGGCATACGAGATCTGATCGTGACTGGAGTTCAGACGT
NG-10
5’CAAGCAGAAGACGGCATACGAGATAAGCTAGTGACTGGAGTTCAGACGT
NG-11
5’CAAGCAGAAGACGGCATACGAGATGTAGCCGTGACTGGAGTTCAGACGT
NG-12
5’CAAGCAGAAGACGGCATACGAGATTACAAGGTGACTGGAGTTCAGACGT

3. Reverse primers for Illumina library construction:

NTS strand

NG-Rev-NTSC
5’AATGATACGGCGACCACCGAGATCTACACTCTTTCCCTACACGACGCTCTTCCGATCTCTGGCAGTACTAGTAAAC
NG-Rev-NTSC+1
5’AATGATACGGCGACCACCGAGATCTACACTCTTTCCCTACACGACGCTCTTCCGATCTNCTGGCAGTACTAGTAAAC

NG-Rev-NTSC+2
5’AATGATACGGCGACCACCGAGATCTACACTCTTTCCCTACACGACGCTCTTCCGATCTNNCTGGCAGTACTAGTAAAC

TS strand

NG-Rev-TSC
5’AATGATACGGCGACCACCGAGATCTACACTCTTTCCCTACACGACGCTCTTCCGATCTCTGTGAACCGGCCCT
NG-Rev-TSC+1
5’AATGATACGGCGACCACCGAGATCTACACTCTTTCCCTACACGACGCTCTTCCGATCTNCTGTGAACCGGCCCT
NG-Rev-TSC+2
5’AATGATACGGCGACCACCGAGATCTACACTCTTTCCCTACACGACGCTCTTCCGATCTNNCTGTGAACCGGCCCT

N = A, C, G or T
